# Can Self-Esteem Protect the Subjective Well-Being of Women in Their 20s from the Effects of Social Media Use? The Moderating Role of Self-Esteem

**DOI:** 10.3390/bs15070964

**Published:** 2025-07-16

**Authors:** Yesolran Kim, Mina Lee

**Affiliations:** Department of Advertising and Public Relations, School of Media and Advertising, Kookmin University, 77 Jeongneung-ro, Seongbuk-gu, Seoul 02707, Republic of Korea; kimysr@kookmin.ac.kr

**Keywords:** self-esteem, social media use, subjective well-being, women in their 20s

## Abstract

This study investigates the relationship between social media use and subjective well-being among South Korean women in their 20s, with a particular emphasis on the moderating role of self-esteem. Cross-sectional data from 611 women in their 20s who had experience using social media platforms was drawn from the Korean Media Panel Survey 2021. A regression analysis revealed that increased social media use was associated with lower subjective well-being. Self-esteem acted as a moderator in the relationship between social media use and subjective well-being. Among individuals with low or medium levels of self-esteem, higher social media use was linked to decreased subjective well-being; however, for those with high self-esteem, social media use did not significantly impact subjective well-being. These findings underscore the significance of self-esteem as a protective factor in the context of social media use and its influence on the subjective well-being among women in their 20s.

## 1. Introduction

The proliferation of social media platforms has directed scholarly attention toward understanding the impact of social media use (hereafter SMU) on psychological outcomes. Existing research reports both positive effects (e.g., increased happiness and life satisfaction, and decreased loneliness) (e.g., Godard & Holtzman, 2024; Karsay et al., 2023; Meng et al., 2023; Murari et al., 2024; Pittman & Reich, 2016) and negative effects (e.g., increased body dissatisfaction, depression, loneliness, social anxiety, and stress, as well as decreased life satisfaction and happiness) (e.g., Azhari et al., 2022; Conte et al., 2024; Karsay et al., 2023; Murari et al., 2024; Satyaninrum et al., 2023; Taylor et al., 2023) of SMU. Given these mixed findings, scholars have increasingly focused on investigating the circumstances under which the effects of SMU vary. While previous studies have shown that the impact of SMU depends on how people engage with social media (e.g., Karsay et al., 2023; Verduyn et al., 2017), some scholars suggest that psychological impact may vary depending on who uses it (Smith et al., 2021). As a result, studies have begun to examine individual factors, such as personality traits, that may moderate the relationship between SMU and mental health and well-being (e.g., Gerson et al., 2016; C. C. Yang, 2016). Among these traits, this study focuses on the role of self-esteem as a potential moderator. Prior research characterizes individuals with high self-esteem as possessing the inherent capacity to nurture, safeguard, and restore their sense of self-worth (Brown et al., 2001). While existing research primarily examines self-esteem as an outcome variable of SMU, finding that SMU negatively influences self-esteem (for a meta-analysis, see Saiphoo et al., 2020), its potential role as a safeguard in mitigating adverse psychological outcomes stemming from SMU remains an important subject of scholarly inquiry.

This study seeks to address the need for further research examining individual factors influencing the relationship between SMU and subjective well-being (hereafter SWB) (Ansari et al., 2024; Smith et al., 2021), and the combined effect of using multiple social media platforms on SWB (Sulaiman et al., 2024; Verduyn et al., 2017). SWB is a vital indicator linked to outcomes such as enhanced health, longevity, and improved social relationships (Diener et al., 2017; Verduyn et al., 2017). To elucidate the complex relationship between SMU, self-esteem, and SWB, the study investigates the moderating role of self-esteem. While extant research has predominantly focused on individual social media platforms, such as Facebook, Instagram, or X (formerly Twitter) (for reviews, Akkas & Turan, 2024; Verduyn et al., 2017), overlooking the broader impact of multi-platform use, this study analyzes overall SMU across various platforms. By analyzing a large secondary dataset from a national survey, it offers a more representative picture of the SMU and its impact on SWB among young adult women. Finally, while many studies have examined individual components of SWB, such as life satisfaction or affect (Ansari et al., 2024), a growing body of research has begun to adopt more comprehensive perspectives by considering multiple facets of SWB in an integrated manner (Jovanović et al., 2024; Suh & Koo, 2011). Building on this perspective, the present study employs an integrative approach grounded in Diener’s (1984) Tripartite Model of SWB, which conceptualizes SWB as comprising three components: life satisfaction, positive affect, and negative affect (Jovanović et al., 2024; Suh & Koo, 2011). By combining these dimensions into a single composite score, this study aims to capture the multidimensional nature of SWB as a holistic evaluation of individuals’ life experiences in a parsimonious and theoretically coherent manner (Suh & Koo, 2011).

This study focuses on women in their 20s in South Korea, offering a framework to explore the relationship between SMU and SWB. With an internet penetration rate exceeding 90% since 2016 and social media usage among younger generations reaching 96%, South Korea represents one of the world’s most digitally connected societies (Statista, 2025a, 2025b). SMU further increased during the COVID-19 pandemic, as restrictions on in-person interaction led many to rely more heavily on online platforms for social connection and communication (Statista, 2023). While South Koreans primarily use globally available platforms (e.g., Instagram and Facebook), there are also regionally popular platforms such as KakaoStory and Naver Band (Statista, 2025d). Although overall SMU rates are higher for males than females in South Korea (Statista, 2025c), young adult females exhibit higher usage rates of Instagram—the most popular social media platform in South Korea—than their male counterparts (Statista, 2025e). When excluding messaging platforms such as KakaoTalk and WhatsApp, and video-sharing platforms like YouTube, social media usage among people in their 20s in South Korea shows patterns similar to those in other countries such as the U.K. and the U.S., where Instagram and TikTok are the most popular platforms, and SMU rates are higher among females than males in their 20s (Korea Press Foundation, 2024; Ofcom, 2024; Pew Research Center, 2024). This environment provides an ideal setting to examine the potential impacts of digital engagement on the well-being of young adult women. Findings from analyzing this demographic may also reflect trends observed or anticipated in other comparable digitized societies. Furthermore, young adult women may face an elevated risk of adverse effects from SMU due to their propensity for social comparison on these platforms (Haferkamp et al., 2012), greater engagement in online social interactions (Ma, 2022), and higher estimated rates of social media addiction (Andreassen, 2015). By focusing on this demographic, this study aims to enrich scholarly discussion on the intersection of technology and well-being, offering global insights to inform interventions that enhance the well-being of young women in the digital era.

Given this, the study explores the influence of SMU on the SWB of women in their 20s, as well as the potential moderating effect of self-esteem. The rich-get-richer and poor-get-poorer hypotheses provide a theoretical basis for understanding the moderating effect of self-esteem. The rich-get-richer hypothesis suggests that individuals with high personal assets, such as self-esteem or extraversion, are more likely to benefit from SMU, since they already possess the social skills necessary to use these platforms in ways that yield greater social benefits (Cheng et al., 2019; Saiphoo et al., 2020; Smith et al., 2021). The poor-get-poorer hypothesis suggests that individuals with limited social capital and negative personal assets, such as low self-esteem, may be more vulnerable to negative psychosocial outcomes of SMU (Frison & Eggermont, 2020; Selfhout et al., 2009; Snodgrass et al., 2018). Drawing upon the “rich-get-richer hypothesis” and “poor-get-poorer hypothesis”, this study examines whether young women with low self-esteem are more susceptible to the negative psychological effects of SMU, while those with high self-esteem are less affected. Understanding these dynamics offers deeper insights into the psychological and emotional outcomes associated with SMU. The study is of considerable academic significance as it explains the inconsistent findings reported in the existing literature by emphasizing the importance of considering individual factors that may influence the effects of SMU. The findings also offer practical insights for policymakers and practitioners seeking to advance the well-being of young women in today’s digital age.

### 1.1. Social Media Use and Subjective Well-Being

With the increasing prevalence of SMU, scholarly attention has increasingly been directed toward examining the relationship between SMU and SWB. SWB, defined as an individual’s cognitive and affective evaluations of their life (Diener et al., 2002), comprises cognitive components, such as life satisfaction, and affective components, such as positive and negative affect (Verduyn et al., 2017). Research on the effects of SMU on SWB has yielded mixed results, with some studies suggesting a positive association between SMU and SWB (e.g., Godard & Holtzman, 2024; Meng et al., 2023; Pittman & Reich, 2016). For instance, a study of 253 undergraduate students in the U.S. found that those using image-based social media platforms such as Instagram and Snapchat reported greater life satisfaction and happiness (Pittman & Reich, 2016). Similarly, a recent study of 1198 adolescents in China showed a positive correlation between mobile SMU and life satisfaction (Meng et al., 2023). A meta-analysis also indicated that active SMU is associated with greater well-being and positive affect (Godard & Holtzman, 2024). However, studies have also reported negative effects of SMU on SWB (e.g., Conte et al., 2024; Tromholt, 2016; Wirtz et al., 2021). Earlier research linked Facebook use to decreased mood, affect, and life satisfaction (Kross et al., 2013; Sagioglou & Greitemeyer, 2014). An experimental study of 1095 Facebook users in Denmark revealed that a one-week break from Facebook use led to increased life satisfaction and more positive emotions compared to the platform’s continued use (Tromholt, 2016). More recently, a study of 77 university students in the U.S. revealed that greater use of various social networking sites (e.g., Facebook, Twitter, and Instagram) correlated with greater negative affect over a 10-day period (Wirtz et al., 2021). A review of 20 studies also reported a negative impact of TikTok use on adolescents’ mental health and life satisfaction (Conte et al., 2024).

To elucidate the complex relationship between SMU and SWB, scholars are increasingly investigating the circumstances under which the effects of SMU differ. This includes contextual factors, such as the types of activities, the nature of uploaded content, the size of social networks, and the social context of SMU (e.g., Karsay et al., 2023; Oh et al., 2014; Reinecke & Trepte, 2014; Vaid et al., 2024). Several studies have examined how specific social media activities impact SWB. Active use, such as self-disclosure (Lee et al., 2011), sharing (S.-S. Wang, 2013), status updates, commenting on content, and writing blogs (J. Wang et al., 2014), posting and chatting (Wenninger et al., 2014), and messaging (Karsay et al., 2023), was positively related to SWB. On the other hand, passive use, such as browsing others’ profiles and news feeds (Wenninger et al., 2014), consuming social information (Krasnova et al., 2015), and general browsing (Karsay et al., 2023), was negatively related to SWB. A critical review of the literature suggests a possible mechanism underlying this relationship—active SMU may increase social capital and feelings of social connectedness, resulting in higher SWB, whereas passive use may increase social comparison and envy, leading to decreased SWB (Verduyn et al., 2017). In addition, the characteristics of posted content, such as the valence of status updates and authenticity of online profiles, have also been shown to affect the relationship between SMU and well-being. For instance, frequently posting negative status updates has been negatively associated with SWB, whereas frequent positive updates show a positive association (Locatelli et al., 2012). In a longitudinal study, authentic self-presentation in online profiles was found to positively influence SWB (Reinecke & Trepte, 2014). Regarding the size of social networks, a meta-analysis of 90 articles revealed a positive association between the number of friends on online social networking sites and well-being (Huang, 2021).

While previous studies have focused mainly on the influence of contextual factors, such as types of social media activities and social network sizes, scholars have turned their attention to the influence of individual factors, especially personality traits, on well-being and mental health (Gerson et al., 2016; C. C. Yang, 2016). For instance, a study of 337 adult participants from the United States and the United Kingdom found that goal-driven persistence moderated the relationship between Facebook social comparison and eudaimonic well-being (a sense of meaning and purpose in life). For those with high levels of goal-driven persistence, negative social comparisons on Facebook were positively associated with eudaimonic well-being (Gerson et al., 2016). Another study of 209 university students in the U.S. showed that social comparison orientation moderated the relationship between Instagram use and loneliness. Specifically, for those with low social comparison orientation, Instagram interactions (e.g., commenting, replying, and tagging) were linked to reduced loneliness (C. C. Yang, 2016). Interestingly, a recent longitudinal study of 1632 young adults in the U.S. examined both contextual and personal factors in the relationship between SMU and well-being (Vaid et al., 2024). This study found that individuals who used social media in certain settings, such as social places and nature, and around certain people, such as family members and close ties, and who had high levels of neuroticism, depression, or loneliness reported more negative well-being after using social media. While these studies suggest that certain personality traits can moderate the impact of SMU on SWB, the current body of research remains limited, necessitating further empirical investigation to deepen our understanding of the potential moderating role of individual personality traits.

### 1.2. Self-Esteem as a Moderator

Among various personality traits, this study focuses on the role of self-esteem as a potential moderator. Self-esteem refers to an individual’s overall evaluation and perception of their own worth (Rosenberg, 1965), shaped by their experiences and social interactions (Bukhari et al., 2023). Research suggests that self-esteem can be viewed both as a trait (or global self-esteem) that remains stable and consistent over time and as a state self-esteem that is unstable and influenced by situations and events in a given moment (Abdel-Khalek, 2016; Brown et al., 2001). However, for most individuals, self-esteem is generally considered a relatively stable trait (Saiphoo et al., 2020; Trzesniewski et al., 2003). Drawing on the existing literature, this study focuses on trait self-esteem and how it influences the effects of social media on an individual’s well-being. A longitudinal study revealed that self-esteem was prospectively associated with various positive life outcomes and well-being, such as increased levels of job and relationship satisfaction, positive affect, and physical health, as well as decreased levels of negative affect and depression (Orth et al., 2012).

In the literature on social media and self-esteem, self-esteem has mainly been studied as a dependent variable of SMU. A meta-analysis of 121 studies revealed a small, negative relationship between SMU and self-esteem (Saiphoo et al., 2020). However, some studies have also examined the mediating role of self-esteem (e.g., Apaolaza et al., 2013; Bukhari et al., 2023; Chen et al., 2016; S. Yang & Zhang, 2022). For example, in a field study of 466 older Chinese adults, functional SMU (i.e., use of social media for fulfilling life tasks without emotional engagement in others’ posts) indirectly increased loneliness through mediation by cognitive self-esteem (S. Yang & Zhang, 2022). Similarly, a survey of 255 Pakistani college students revealed that self-esteem mediated the positive relationship between WhatsApp use and life satisfaction (Bukhari et al., 2023). Beyond its mediating role, limited research has begun to explore the moderating role of self-esteem in the relationship between SMU and psychological outcomes (e.g., Choi, 2019). For instance, an online survey involving 107 Korean female college students found that increased passive Instagram use was associated with higher levels of depression among those with lower self-esteem than those with higher self-esteem (Choi, 2019). However, this study provides limited evidence on the potential moderating role of self-esteem due to its focus on a single platform (Instagram) and measurement of only one aspect of psychological impact (depression). The scarcity of research and empirical evidence on the moderating role of self-esteem in the impact of SMU on SWB highlights the need for further research in this area.

The rich-get-richer hypothesis and the poor-get-poorer hypothesis inform the moderating role of self-esteem in the relationship between SMU and SWB (Kraut et al., 2002; Smith et al., 2021). According to the rich-get-richer hypothesis and the poor-get-poorer hypothesis, individuals with positive personal assets such as high self-esteem or extraversion are more likely to benefit from SMU due to their stronger social skills (Cheng et al., 2019; Saiphoo et al., 2020; Smith et al., 2021), whereas those with low self-esteem or limited social capital may be more susceptible to the negative effects of SMU and experience further declines in well-being (Frison & Eggermont, 2020; Selfhout et al., 2009; Snodgrass et al., 2018). Consistent with these hypotheses, research has shown that extroverts’ SMU is associated with increased online social capital accrual, while introverts do not gain the same benefits (Cheng et al., 2019; Moshkovitz & Hayat, 2021). Likewise, Valkenburg et al. (2017) found that individuals with high self-esteem engaged in more SMU, which in turn enhanced their self-esteem further. Conversely, for adolescents with low perceived friendship quality, internet use for non-communication purposes (i.e., surfing) reported higher levels of depression and social anxiety (Selfhout et al., 2009).

Social Comparison Theory provides a promising explanation for why individuals with low self-esteem may be more vulnerable to the negative effects of SMU. According to Social Comparison Theory, people tend to evaluate their own self-worth and abilities in comparison to others, and the social media environment may facilitate this process, often resulting in negative psychological outcomes (Saiphoo et al., 2020; Sulaiman et al., 2024). Several studies suggest that prolonged exposure to embellished or amplified depictions of others on social media may give rise to tendencies of upward social comparisons and feelings of envy, consequently diminishing an individual’s SWB (Krasnova et al., 2015; Verduyn et al., 2017). As suggested by the literature on Social Comparison Theory, individuals with low self-esteem tend to engage in frequent upward social comparisons while using social media, which may exacerbate the detrimental effects of SMU (Eraslan-Capan, 2015; Saiphoo et al., 2020). Also, those with low self-esteem—often characterized by low confidence, self-deprecating thoughts, and a pessimistic outlook—are likely to engage in problematic SMU (e.g., posting negative content and lurking on others’ content) and receive fewer positive responses and social support, which may contribute to worsening psychological outcomes (Forest & Wood, 2012; Liu & Baumeister, 2016; Locatelli et al., 2012; Saiphoo et al., 2020).

In addition, the affective model of self-esteem development and the risk-buffering hypothesis support the idea of the protective role that self-esteem may play in mitigating negative effects of SMU (Brown et al., 2001; Luthar et al., 2015; P. Wang et al., 2018). The affective model of self-esteem development posits that while self-esteem initially emerges as a reaction to relational and temperamental factors, once established, individuals with high self-esteem possess the capacity to foster, safeguard, and restore their sense of self-value (Brown et al., 2001). The risk-buffering hypothesis suggests that positive individual attributes such as self-esteem can mitigate the negative influence of risk factors on psychological outcomes (Luthar et al., 2015; P. Wang et al., 2018). Thus, individuals with high self-esteem are likely to employ diverse strategies to safeguard and restore their sense of self-value, when encountering negative experiences on social media (Brown et al., 2001; Leary & Baumeister, 2000; Luthar et al., 2015; P. Wang et al., 2018). Further, individuals with high self-esteem—often characterized by high confidence and positive attitudes toward life—tend to engage in active and positive SMU and are more likely to receive positive feedback, which can enhance the psychological benefits of SMU (Mann et al., 2004; Saiphoo et al., 2020; Smith et al., 2021; Valkenburg et al., 2017).

Therefore, building on these hypotheses and relevant literature, this study proposes that young adult women with high self-esteem are likely to experience fewer adverse and more positive effects of SMU on subjective well-being, whereas those with low self-esteem may be more susceptible to negative effects.

### 1.3. Aims of Study and Research Questions

**Figure 1:**
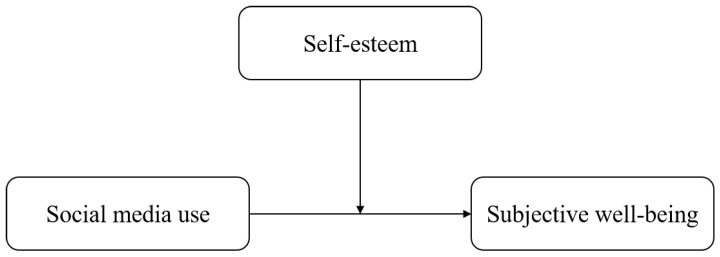
Research model.

Given that the existing literature suggests the negative effects of SMU may be more prevalent or impactful among young women and given the limited understanding of the moderating role of self-esteem in the SMU-SWB relationship (Andreassen, 2015; Choi, 2019; Fabio & Tripodi, 2024; Haferkamp et al., 2012), further investigation is needed to explore these complex dynamics. Therefore, this study examines the influence of SMU on the SWB of women in their 20s in South Korea and investigates the moderating role of self-esteem in this relationship. It seeks to evaluate whether self-esteem serves as a safeguard against the potential adverse effects of SMU. Accordingly, the study answers the following research questions (See Figure 1):

RQ1: Does SMU increase or decrease the SWB of South Korean women in their 20s?
RQ1-1: Is SMU positively associated with SWB of South Korean women in their 20s?
RQ1-2: Is SMU negatively associated with SWB of South Korean women in their 20s?
RQ2: Does the impact of SMU on SWB vary based on the level of self-esteem among South Korean women in their 20s?
RQ2-1: Among individuals with high levels of self-esteem, is higher SMU associated with higher SWB (rich-get-richer hypothesis)?
RQ2-2: Among individuals with low levels of self-esteem, is higher SMU associated with lower SWB (poor-get-poorer hypothesis)?

## 2. Methods

### 2.1. Data Overview

This study used cross-sectional data from the Korean Media Panel Survey 2021 (KMPS 2021), a nationally approved statistical project (National Approved Statistics No. 405001 in South Korea) conducted by the Korea Information Society Development Institute, a South Korean government-funded research institute. The KMPS 2021 employed stratified quota sampling based on the national population and housing census as the sampling frame to draw a nationally representative sample of 10,800 from 17 cities in South Korea. Trained interviewers conducted computer-assisted personal interviews in each household over approximately nine weeks from June to July 2021, resulting in 10,154 complete responses (response rate: 94.02%) (Korea Information Society Development Institute, 2022; complete dataset available at the provided URL).

The study selected cases relevant to the research topic from this dataset. The selection criteria included women in their 20s who reported using social media platforms like Instagram, Facebook, TikTok, or X (formerly Twitter). Applying the three filtering criteria—(1) individuals aged 20 to 29 (1274 out of 10,154), (2) women (651 out of 1274), and (3) individuals with experience using social media platforms (611 out of 651)—resulted in a final sample of 611 responses.

### 2.2. Measures

*Social media use.* This study defined SMU as the average daily time spent using platforms such as Instagram, Facebook, TikTok, and X (formerly Twitter). The KMPS 2021 asked respondents, “How much time do you spend on social media on average each day?” and instructed them to report their usage time in numerical form for both weekdays and weekends. For analysis, these usage times were converted into minutes, summed, and then averaged to represent daily SMU. A higher mean value indicates greater daily time spent on social media platforms. The sample’s mean daily SMU was 71.016 min (*SD* = 83.000).

*Subjective well-being*. The KMPS 2021 measured SWB using the Concise Measure of Subjective Well-Being (COMOSWB) developed by Suh and Koo (2011). This measure, grounded in Diener’s (1984) Tripartite Model of SWB, is based on items from the Satisfaction with Life Scale (SWLS; Diener et al., 1985) and the Scale of Positive and Negative Experience (SPANE; Diener et al., 2010), and was designed to capture both universal and culturally specific aspects of the SWB construct. Given that administering the full SWLS and SPANE would involve a relatively large number of items, the COMOSWB was developed as a brief, culturally adapted measure that reflects the core elements of SWB in the Korean context (Suh & Koo, 2011). The scale includes three SWB components—life satisfaction, positive affect, and negative affect—assessed through nine items on a 7-point Likert scale. These items reflect life satisfaction (personal, relational, and collective aspects), positive affect (pleased, happy, and comfortable), and negative affect (irritated, negative, and lethargic). The SWB index was calculated by subtracting the sum of responses to the three negative affect items from the combined sum of responses to the three life satisfaction items and the three positive affect items. This was then converted into a percentage, yielding a theoretical range of 0 to 100 (Suh & Koo, 2011), where higher scores indicated greater SWB. For the analysis, SWB scores were calculated using this process (Cronbach’s alpha = 0.870).

*Self-esteem.* KMPS 2021 measured self-esteem using a Korean translation of Rosenberg’s (1965) Self-Esteem Scale (RSES), a widely used measure of trait self-esteem. Respondents were presented with the following prompt: “Below is a list of statements related to thoughts about oneself. Please indicate the number that corresponds to your feelings for each statement”. They were asked to respond to 10 items using a 4-point Likert scale (1 = *not at all*, 4 = *always*). Example items include: “I feel that I have a number of good qualities”, “I take a positive attitude toward myself”, “At times I think I am no good at all”, and “I wish I could have more respect for myself”. Items with reverse meanings were reverse-scored to ensure consistency, and higher average scores indicated higher self-esteem. The mean value of the sum of all items was calculated for the analysis (Cronbach’s alpha = 0.741; ≥0.70 is considered acceptable; Tavakol & Dennick, 2011).

*Control variables*. Scholars have demonstrated that various sociodemographic characteristics—such as age, education, income, employment status, and marital status—are associated with SWB (Song & Lee, 2022). These factors are important because they can significantly shape how individuals experience and report their overall well-being. Therefore, the present study controlled for these variables in order to isolate the unique effects of SMU and self-esteem on SWB and to provide a clearer understanding of the relationships among the research variables.

Age was calculated by subtracting the respondent’s reported year of birth in the KMPS 2021 from the survey year, 2021.

Education was measured in the KMPS 2021 using the following categories: no formal education, elementary school graduate, middle school graduate, high school graduate, university graduate, and graduate school or higher. As no respondents in the sample fell into the “no formal education” or “elementary school graduate” categories, the analysis included education levels from “middle school graduate” to “graduate school or higher”.

Income was measured as the respondent’s average monthly pre-tax income, divided into seven categories in Korean Won (KRW): no income; less than 500,000; 500,000 to under 1,000,000; 1,000,000 to under 2,000,000; 2,000,000 to under 3,000,000; 3,000,000 to under 4,000,000; and 4,000,000 or more. These income levels were used in the analysis.

Employment status was assessed using the question, “Do you currently have a job?” with *Yes* or *No* responses. For analysis, *No* was coded as unemployed and *Yes* as employed, with unemployed as the reference category for dummy coding.

Marital status was measured with the following categories: single, married, widowed, and divorced. For analysis, these categories were recategorized into, currently unmarried (including single, widowed, and divorced) and currently married, with currently unmarried serving as the reference category for dummy coding.

### 2.3. Data Analysis

Data analysis was conducted using SPSS version 26.0 and Hayes’s (2017) PROCESS macro 4.2. Descriptive statistics, including frequencies, means, and standard deviations, were calculated using SPSS to examine the sample’s sociodemographic characteristics and key measures. To answer the research question investigating the relationship between SMU and SWB and the moderating effect of self-esteem among women in their 20s, Model 1 of the PROCESS macro was employed.

## 3. Results

### 3.1. Sociodemographic Characteristics of Respondents

Table 1 presents the sociodemographic characteristics of the sample. The average age of respondents was 24.60 years (*SD* = 2.838), with the most common age being 26 years (12.439%) and 25 years (11.948%). In terms of education, the majority of respondents (91.489%) were university graduates, followed by high school graduates (7.038%). Only a tiny percentage of respondents (1.309%) had completed graduate school or higher. Income distribution showed that the largest proportion of respondents (45.008%) reported no income. Among those with income, the majority fell within the monthly income range of KRW 2,000,000 to KRW 2,999,999 (27.332%). Nearly half of the respondents (48.936%) were employed, while the remaining (51.064%) were unemployed. Finally, regarding marital status, the vast majority (98.036%) were currently unmarried (including single, widowed, or divorced), with only a small percentage (1.964%) currently married.

### 3.2. Descriptive Statistics and Variable Correlations

Table 2 presents the descriptive statistics and correlations among the variables. Among South Korean women in their 20s, the mean values for SMU, SWB, and self-esteem were 71.016 (*SD* = 83.000), 69.325 (*SD* = 13.651), and 3.065 (*SD* = 0.400), respectively. The analysis revealed a significant negative correlation between SMU and SWB (*r* = −0.140, *p* < 0.05) and a positive correlation between SWB and self-esteem (*r* = 0.578, *p* < 0.05).

### 3.3. Association Between Social Media Use and Subjective Well-Being and Moderating Effects of Self-Esteem

A regression model was employed to analyze the relationship between SMU, SWB, and self-esteem, with SMU as the independent variable, SWB as the dependent variable, and self-esteem as the moderator. Additionally, control variables included sociodemographic characteristics, such as age, education, income, employment status, and marital status. The overall model accounts for 35.5% of the variance in SWB (*R*^2^ = 0.355, *p* < 0.05; refer to Table 3 for details).

The regression analysis yielded several key findings. First, SMU was found to have a significant negative effect on SWB (*B* = −0.112, *SE* = 0.039, *t* = −2.862, *p* < 0.05), suggesting that greater social media engagement is associated with lower levels of SWB. Second, self-esteem showed a positive association with SWB (*B* = 17.126, *SE* = 1.528, *t* = 11.208, *p* < 0.05), indicating that higher self-esteem is linked to greater SWB. Notably, the interaction between SMU and self-esteem was found to be significant (*B* = 0.030, *SE* = 0.013, *t* = 2.373, *p* < 0.05), highlighting the moderating effect of self-esteem on the relationship between SMU and SWB. Additionally, the control variables—age, education, income, employment status, and marital status—were not significant predictors of SWB in this model.

**Figure 2:**
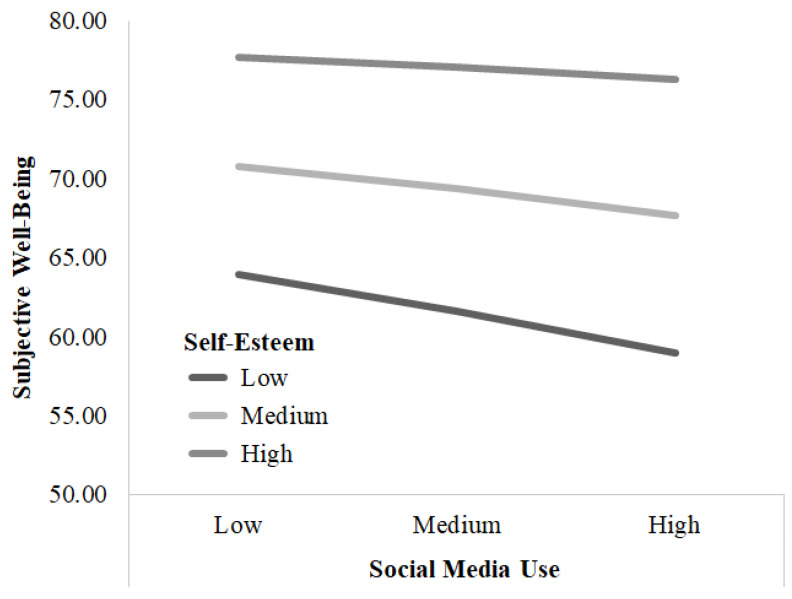
The moderating effect of self-esteem on social media use and subjective well-being.

The regression analysis revealed significant conditional effects of self-esteem on the relationship between SMU and SWB. As shown in Table 4 and Figure 2, SMU had a significant negative effect on SWB for individuals with low levels of self-esteem (low: 1 *SD* below the mean; *B* = −0.033, 95% CI [−0.048~−0.018]) and those with medium levels of self-esteem (medium: mean; B = −0.021, 95% CI [−0.031~−0.010]). This indicates that increased SMU is associated with lower SWB among individuals with average and below-average levels of self-esteem. However, for those with high levels of self-esteem (high: 1 *SD* above the mean), the effect of SMU on SWB was not significant (B = −0.009, 95% CI [−0.023~0.006]). This suggests that individuals with higher self-esteem may be less affected by SMU in terms of their SWB.

Furthermore, the Johnson–Neyman technique revealed a negative correlation between SMU and SWB for individuals with a self-esteem score below 3.338 (*B* = −0.013, 95% CI [−0.025~−0.001]), representing 75.777% of the sample. However, when the self-esteem score reached 3.338 or higher, no significant association was observed (*B* = −0.012, 95% CI [−0.025~0.000]), representing 24.223% of the sample. These findings emphasize the importance of considering specific levels of self-esteem when examining the relationship between SMU and SWB.

## 4. Discussion

This study investigated the relationship between SMU and SWB among women in their 20s in South Korea, with a particular focus on the moderating role of self-esteem. The findings reveal a negative association between SMU and SWB and a positive association between self-esteem and SWB, even after controlling for sociodemographic characteristics. Notably, self-esteem significantly moderated the relationship between SMU and SWB. Specifically, for individuals with low to moderate levels of self-esteem, SMU was negatively associated with SWB. However, no significant association was observed between SMU and SWB for those with high levels of self-esteem. The analysis identified a self-esteem score of 3.338 as the threshold at which the significance of this relationship changed. The findings offer valuable insights into how SMU relates to SWB among women in their 20s and how self-esteem levels influence this relationship.

This study found a negative association between SMU and SWB among women in their 20s—those who spent more time on social media reported lower levels of SWB. The significant negative association between SMU and SWB suggests that excessive use of social media platforms may be linked to lower levels of SWB. While social media platforms provide opportunities for connection, interaction, and information sharing, they can also expose users to negative experiences, such as the overrepresentation of others’ positive life events and the resulting social comparisons (Najam, 2023; Wirtz et al., 2021). As people tend to post content on social media that conveys primarily favorable impressions, individuals may mistakenly conclude that others’ lives consist predominantly of positive experiences, unlike their own, which include both positive and negative moments (Wirtz et al., 2021). Consequently, increased time spent on social media may heighten the contrast effect and upward social comparison between one’s own life and the idealized lives depicted on social media, leading to lower SWB (Krasnova et al., 2015; Saiphoo et al., 2020; Sulaiman et al., 2024; Verduyn et al., 2017). Although previous studies have yielded mixed findings on the relationship between SMU and SWB (e.g., Murari et al., 2024; Verduyn et al., 2017), this study aligns with the growing body of literature suggesting that excessive SMU can adversely affect mental health and well-being (Conte et al., 2024; Kross et al., 2013; Sagioglou & Greitemeyer, 2014; Tromholt, 2016; Wirtz et al., 2021), providing further empirical evidence to support this scholarly view.

The study also found that self-esteem was positively associated with SWB among women in their 20s. Those with higher levels of self-esteem reported greater SWB, suggesting that self-esteem may serve as a psychological resource that promotes well-being. High self-esteem can provide individuals with confidence and a positive attitude toward life (Saiphoo et al., 2020), enabling them to cope with negative experiences and recognize the positive aspects of their lives (Mann et al., 2004). This, in turn, can contribute to greater SWB. In contrast, low self-esteem is associated with a lack of confidence and self-deprecating attitudes (Saiphoo et al., 2020), making individuals more vulnerable to negative experiences and potentially undermining well-being. This underscores the importance of self-esteem as a fundamental determinant of well-being, suggesting that individuals with higher self-esteem may possess greater resilience against factors that could negatively affect their SWB. This is consistent with a body of research demonstrating a positive association between self-esteem and well-being (e.g., Emmons & Diener, 1985; Kong et al., 2015).

Finally, this study identified self-esteem as a moderator in the relationship between SMU and SWB among women in their 20s. For those with low or moderate levels of self-esteem (scores below 3.338 on a 4-point scale), increased SMU was associated with a decline in SWB (effect = −0.033 at low self-esteem and −0.021 at moderate self-esteem). However, for those with high self-esteem (score of 3.338 or higher), SMU was not significantly associated with SWB (effect = −0.009, n.s.). Considering that the self-esteem scale ranged from 1 (“not at all”) to 4 (“always”), a score of 3.338 roughly corresponds to a response pattern between “mostly” (3) and “always” (4), indicating a relatively high level of self-esteem. The moderating effect of self-esteem suggests that individuals with higher self-esteem may be more resilient to potential adverse effects of social media on SWB. This effect likely stems from their capacity to effectively safeguard and restore their self-worth, as expounded upon by Brown et al. (2001). Alternatively, it is also possible that those with high self-esteem engage with social media in healthier ways, thereby avoiding situations that could negatively affect their well-being. For instance, individuals with high self-esteem are better equipped to focus on the positive aspects of social interactions (Mann et al., 2004; Saiphoo et al., 2020), which may help them use social media in a more adaptive manner. In contrast, individuals with lower self-esteem appear more vulnerable to experiencing a stronger negative impact on their well-being from increased SMU. This finding is consistent with the poor-get-poorer hypothesis (Frison & Eggermont, 2020; Selfhout et al., 2009; Snodgrass et al., 2018). Some possible explanations from prior research may account for the negative association between SMU and SWB among individuals with low self-esteem. Studies have shown that individuals with low self-esteem post more negative updates, often receiving fewer likes than those with high self-esteem (Forest & Wood, 2012). In another study involving 251 college students found a negative correlation between frequently posting negative status updates on Facebook and SWB (Locatelli et al., 2012). These studies suggest that individuals with low self-esteem may be more likely to share negative content, eliciting less favorable responses from others, which, in turn, could contribute to lower positive affect and life satisfaction. Another explanation stems from research showing that individuals with low self-esteem tend to engage in more social comparisons while using social media, exacerbating the negative impact of SMU (Eraslan-Capan, 2015; Saiphoo et al., 2020). For instance, an experimental study (Jones & Buckingham, 2005) found that female students with low self-esteem exhibited lower body esteem when exposed to a picture of an attractive woman (upward comparison) compared with a picture of an unattractive woman (downward comparison). Drawing on these studies, it can be inferred that extended exposure to idealized portrayals on social media platforms may amplify upward comparison tendencies among young adult women with lower self-esteem, consequently undermining their SWB. Further research is needed to elucidate these possible mechanisms underlying the moderating effects of self-esteem observed in this study.

This study makes several significant scholarly and practical contributions. First, it expands scholarly understanding of the complex relationship between SMU and well-being by focusing on the experiences of South Korean women in their 20s. The observed negative effect of SMU on SWB underscores the importance of cultivating healthier social media usage habits, while the positive association between self-esteem and SWB highlights the crucial role that individual self-perception plays in shaping overall well-being. Furthermore, the moderating effect of self-esteem emphasizes its potential protective function in mitigating the negative impact of SMU. In an era when young adult women spend considerable time on social media platforms (Andreassen, 2015; Statista, 2025b) and remain particularly vulnerable to their adverse effects (Haferkamp et al., 2012), this study, by examining the complex interplay between SMU, SWB, and self-esteem in this demographic, enriches our understanding and advances scholarly discourse on the well-being of young women in the digital age.

Second, this study addresses the conflicting findings regarding the relationship between SMU and well-being (Murari et al., 2024; Verduyn et al., 2017) by highlighting the moderating role of self-esteem. The findings demonstrate that the relationship between SMU and SWB varies according to an individual’s level of self-esteem. Among women in their 20s, those with low or moderate self-esteem showed a negative association between time spent on social media and SWB, whereas no significant association was observed for those with high self-esteem. These findings suggest that the relationship between SMU and SWB is not uniform but rather contingent on individuals’ perceptions of their self-worth. The study holds scholarly significance, as it provides a comprehensive and compelling explanation for the inconsistent findings reported in prior research. Furthermore, the findings align with academic perspectives emphasizing the importance of considering individual differences, such as self-esteem, when examining the psychological outcomes of SMU (Choi, 2019; Ellison et al., 2007; Saiphoo et al., 2020; Steinfield et al., 2008).

Third, the findings highlight the opportunity to develop targeted intervention programs tailored to women in their 20s, taking into account varying self-esteem levels and SMU to improve SWB. For policymakers and practitioners such as psychologists, counselors, social workers, educators, and digital well-being experts, these findings underscore the need for customized strategies that address the distinct circumstances of young women with low or moderate self-esteem, who are particularly vulnerable to negative effects of excessive SMU. Interventions for this group could prioritize building self-esteem, fostering emotional resilience, and enhancing digital literacy. These programs could include workshops designed to enhance self-worth, promote healthy coping strategies for managing online interactions, and educate young women about the psychological risks associated with SMU. Digital literacy education can be particularly valuable, equipping young women to critically engage with online content, minimize the impact of negative feedback, and mitigate harmful social comparisons. By teaching conscious and effective SMU, these interventions can support healthier online engagement. In addition to bolstering self-worth, interventions could focus on managing time spent on social media, setting digital boundaries (e.g., limiting screen time), and encouraging offline activities that promote a positive self-image. For women with high self-esteem, interventions could focus on reinforcing positive self-perceptions and encouraging mindful SMU to ensure their online engagement continues to support their well-being. Furthermore, social media platforms can contribute to enhancing well-being by creating healthier online environments. This could involve integrating features that support self-esteem (e.g., positive feedback systems) while reducing the visibility of content that promotes harmful social comparison. By implementing these targeted, multi-faceted approaches, stakeholders can collaboratively work to enhance the well-being of young women in the digital era.

While this study offers valuable insights, its findings should be interpreted cautiously. The study employed a cross-sectional research design, which allows for examining multiple variables at a single point in time, providing a snapshot of relationships. However, this limits the ability to infer causal relationships among variables (Setia, 2023). It is possible that individuals with poorer SWB may use social media more often as a coping strategy (Zhang et al., 2022). This potential reverse relationship was not examined in this study. Therefore, future research should consider using longitudinal studies or experimental designs to explore reciprocal or bidirectional dynamics between SMU and SWB. Furthermore, this study utilized data from the KMPS 2021. Although the sample was purposively selected, it may not fully capture the diversity of women in their 20s, including variations in experiences, backgrounds, and regional or cultural differences. These factors could influence the relationships between SMU, SWB, and self-esteem, potentially introducing bias. Future research could employ more diverse sampling strategies or conduct subgroup analyses to gain deeper insights. Moreover, it would be valuable to include male participants or adopt a gender-comparative approach, as men are also increasingly affected by social-media-related pressures and social comparison (Boursier & Gioia, 2022). Another limitation is that SMU was measured solely based on average daily usage time due to the use of secondary data, which overlooks the diversity of SMU activities, such as active and passive use. Future studies should employ customized measures to better capture various aspects of SMU. Additionally, there is a need to take into account cultural differences in self-esteem, as research suggests that individuals from East Asian cultures tend to exhibit lower levels of self-esteem compared with those from Western societies (Abdel-Khalek, 2016; Heine & Lehman, 2004). Therefore, future studies could examine whether the moderating effects of self-esteem identified in this study are applicable in Western or other cultural contexts. Finally, studies have also found that self-esteem may mediate the impact of SMU on SWB (Apaolaza et al., 2013; Bukhari et al., 2023; Chen et al., 2016; S. Yang & Zhang, 2022). Future studies investigating whether self-esteem both mediates and moderates the relationship between SMU and SWB can further illuminate the complex interplay among these variables.

## 5. Conclusions

This study provides a more nuanced understanding of the relationship between SMU and SWB among women in their 20s, demonstrating that self-esteem significantly moderates this relationship. The findings suggest that among individuals with low to moderate self-esteem, higher SMU is associated with lower SWB, whereas this association is not observed among those with high self-esteem. To the best of the authors’ knowledge, this study represents the first effort to directly investigate the moderating role of self-esteem in the relationship between SMU and SWB in this demographic. Given that young adult women frequently engage in online social interactions (Ma, 2022), are more prone to social comparison on social media platforms (Haferkamp et al., 2012), and exhibit higher rates of social media addiction (Andreassen, 2015), this study offers valuable insights into the scholarly discourse surrounding the relationship between SMU and well-being in this population. While prior research on the relationship between SMU and SWB has yielded mixed results, this study emphasizes the crucial role of self-esteem in shaping this relationship. The findings not only deepen our understanding of this relationship among young women but also provide valuable insights for researchers, policymakers, and practitioners seeking to promote their well-being in the digital era. Future research addressing the limitations of this study will further refine our understanding of these critical relationships.

## Figures and Tables

**Table 1:** Sociodemographic characteristics of respondents.

Variables	*N*	Percent or *M* (*SD*)
**Age**		24.60 (2.838)
**Education**		
Middle school graduation	1	0.164
High school graduation	43	7.038
University graduation	559	91.489
Graduate school or higher	8	1.309
**Income (** **KRW)**		
No income	275	45.008
Less than 500,000	20	3.273
500,000 to under 1,000,000	24	3.928
1,000,000 to under 2,000,000	98	16.039
2,000,000 to under 3,000,000	167	27.332
3,000,000 to under 4,000,000	23	3.764
4,000,000 or more	4	0.655
**Employment status (ref. unemployed)**		
Employed	299	48.936
Unemployed	312	51.064
**Marital status (ref. currently unmarried)**		
Currently unmarried (single, widowed, or divorced)	599	98.036
Currently married	12	1.964

*N* = 611.

**Table 2:** Descriptive statistics and correlation coefficients of variables.

Variable	*M*	*SD*	1	2	3
1. SMU	71.016	83.000	1		
2. SWB	69.325	13.651	−0.140 *	1	
3. Self-esteem	3.065	0.400	−0.031	0.578 *	1

*N* = 611. * *p* < 0.05.

**Table 3:** The effect of social media use on subjective well-being and moderating effect on self-esteem.

Variables	*B*	*SE*	*t*	95% CI	
				LLCI	ULCI
Age	−0.029	0.215	−0.135	−0.451	0.393
Education	0.723	1.574	0.460	−2.369	3.815
Income	0.286	0.663	0.431	−1.016	1.588
Employment status (ref. unemployed)	−0.296	2.485	−0.522	−6.176	3.583
Marital status (ref. currently unmarried)	1.322	3.271	−0.404	−7.745	5.101
SMU	−0.112	0.039	−2.862 *	−0.189	−0.035
Self-esteem	17.126	1.528	11.208 *	14.125	20.127
Interaction	0.030	0.013	2.373 *	0.005	0.055
*R* ^2^	0.355 *				
Incremental *R*^2^	0.006 *				
*F* (*df*1, *df*2)	41.452 (8, 602)				

*N* = 611. * p < 0.05.

**Table 4:** Conditional effects of self-esteem on the relationship between social media use on subjective well-being.

Value of Self-Esteem	Effect	*SE*	*t*	95% CI	
				LLCI	ULCI
2.665 (Low; Mean −1*SD*)	−0.033	0.008	−4.290 *	−0.048	−0.018
3.065 (Medium; Mean)	−0.021	0.006	−3.765 *	−0.031	−0.010
3.465 (High; Mean +1*SD*)	−0.009	0.007	−1.193	−0.023	0.006

*Note. N* = 611. * p < 0.05.

## Data Availability

The data that support the current findings are available for research purposes via the KISDI STAT, a statistical portal (https://stat.kisdi.re.kr/kor/contents/ContentsList.html?subject=MICRO10&sub_div=D; accessed on 30 September 2022) operated by the Korea Information Society Development Institute (https://www.kisdi.re.kr; accessed on 30 September 2022).
